# Clinical and Genetic Spectrum of Nine Cases of NLRP3-Associated Autoinflammatory Disease (NLRP3-AID) and Identification of One Novel NLRP3 Mutation by Genetic Variation Analyses

**DOI:** 10.1155/2024/5722548

**Published:** 2024-03-06

**Authors:** Yaoyao Shangguan, Xingru Ding, Le Ma, Yi-Xin Cai, Shulei Xiang, Xiu-Feng Huang, Yunyan Shen, Hai-Guo Yu, Wenjie Zheng

**Affiliations:** ^1^Department of Pediatric Rheumatology, The Second Affiliated Hospital and Yuying Children's Hospital of Wenzhou Medical University, Wenzhou, Zhejiang, China; ^2^Key Laboratory of Structural Malformations in Children of Zhejiang Province, Wenzhou, Zhejiang, China; ^3^Zhejiang Provincial Clinical Research Center for Pediatric Disease, The Second Affiliated Hospital and Yuying Children's Hospital of Wenzhou Medical University, Wenzhou, Zhejiang, China; ^4^Rheumatology and Immunology Department, Children's Hospital of Nanjing Medical University, Nanjing, China; ^5^Department of Nephrology and Immunology, Children's Hospital of Soochow University, Suzhou, China

## Abstract

**Purpose:**

NLRP3-associated autoinflammatory disease (NLRP3-AID) is characterized by gain-of-function variants in the NLRP3 gene. Since there are little literature focusing on pediatric NLRP3-AID in China, we aimed to elucidate the phenotypic and genotypic profiles of Chinese patients with NLRP3-AID.

**Methods:**

Patients with NLRP3-AID at three rheumatology centers in China were genotyped through whole exome sequencing or gene panel sequencing. Sanger sequencing was performed on all patients and their parents. Clinical phenotype, treatment, and prognosis were analyzed.

**Results:**

Nine patients with NLRP3-AID were enrolled between December 2014 and October 2022 with an average follow-up period exceeding 30 months. The median age of onset was 12 months, and 66.7% were younger than 3 years old. The diagnosis was significantly delayed and the median delay duration was 115 months. The patients most commonly presented with rash (100%), arthritis/arthralgia (88.9%), lymphadenopathy (88.9%), fever (77.8%), and growth retardation (44.4%). During acute attack, white blood cell, C-reactive protein, and/or erythrocyte sedimentation rate all increased in all cases, and inflammatory markers remained elevated beyond 7 days postfever resolution in 57.1% of patients (4/7). Two cases of chronic infantile neurological cutaneous articular syndrome (CINCA) had clubbed fingers, one with interstitial lung disease, a finding rarely reported. Treatment with glucocorticoids (77.8%) and biologic agents (33.3%) yielded 66% complete remission and 33% partial remission. Genetic analysis identified eight pathogenic NLRP3 missense mutations, including one novel mutation.

**Conclusions:**

Our study illuminated the distinct clinical and genetic features of Chinese NLRP3-AID patients, emphasizing the significance of early genetic screening. Despite delayed diagnosis, treatment primarily with glucocorticoids and biologic agents, led to favorable outcomes. Genetic heterogeneity, including a novel mutation, highlighted the complexity of NLRP3-AID in this population.

## 1. Introduction

NLRP3-associated autoinflammatory disease (NLRP3-AID) is a spectrum of autosomal dominant inherited inflammasome diseases caused by gain-of-function mutations in the NLRP3 gene encoding cryopyrin and is previously known as cryopyrin-associated periodic syndromes (CAPS) [1]. NLRP3-AID comprises three distinct presentations with increasing severity: familial cold autoinflammatory syndrome (FCAS), Muckle–Wells syndrome (MWS), and neonatal-onset multisystem inflammatory disorder (NOMID), also known as chronic infantile neurological cutaneous articular syndrome (CINCA) [2]. The estimated prevalence of NLRP3-AID is 1–2 in 1 million in USA [3] and 1 in 0.36 million in France [4].

The clinical manifestations of NLRP3-AID are heterogeneous, characterized by self-subsiding fever and rash in mild cases, while hydrocephalus and cognitive impairment in severe cases. FCAS is typically presented with urticarial-like rash, conjunctivitis, and fever following generalized cold exposure, which is the mildest form of NLRP3-AID [2, 3]. MWS, first reported by Muckle and Wells [4] in 1962, shares many similarities with FCAS but not triggered by cold exposure. MWS is further marked by the presence of sensorineural deafness and amyloidosis [4]. As for CINCA, it is the severest condition of NLRP3-AID distinguished by chronic severe urticarial-like rash, arthritis fever with significant central nervous system involvement, and distal femur arthropathy [5].

The human NLRP3 gene is located on chromosome 1q44 consisting of nine exons [6] and mainly functions in the assembly of the NLRP3 inflammasome. Gain-of-function mutations of NLRP3 gene lead to unexpected activation of the inflammasome and further result in overexpression of proinflammatory cytokine IL-1*β*, therefore triggering systemic inflammation [2]. Since its initial report in 2001 [7], more than 260 variants have been identified in NLRP3-AID patients.

Given the complexity of clinical presentations and genetic diversity, our study addresses the paucity of research on pediatric NLRP3-AID in China. With a cohort of nine children and an average follow-up period exceeding 30 months, our aim is to provide new clinical and genetic insights, contributing to early disease identification and improved recognition in the Chinese pediatric population.

## 2. Methods

### 2.1. Study Subjects

The study protocol was approved by the Institutional Review Board and the Medical Ethics Committee of The Second Affiliated Hospital and Yuying Children's Hospital of Wenzhou Medical University (2021-K-327-02). The informed consent was obtained from the parents or patients (if more than 18 years of age). The inclusion criteria were the presence of a confirmatory NLRP3 genotype and at least two of the following five typical symptoms [8]: (1) urticarial rash, (2) cold/stress-triggered episodes, (3) sensorineural hearing loss, (4) chronic aseptic meningitis, and (5) skeletal abnormalities (epiphysial overgrowth/frontal bossing). Standardized case report form was used to record the demographic information, genetic results, and clinical features including symptoms, physical examinations, laboratory tests, and treatments.

The therapeutic outcomes were classified into complete remission (CR), partial remission (PR), and no remission (NR). NR was defined as no improvement in inflammatory markers or improvement less than 50%, worsening of symptoms, or increased organ involvement. PR was defined as an improvement of ≥50% in inflammatory markers and symptom improvement with no increase in organ involvement. CR was defined as normal inflammatory markers, disappearance of symptoms, no increase in organ involvement, and no active manifestations.

### 2.2. Identification of Genetic Variants

DNA samples were extracted from peripheral blood of patients and their parents. High-throughput sequencing was carried out to detect variants of NLRP3 (NM_004895). The suspected variants would be further investigated in HGMD and OMIM databases. Pathogenicity predictions and conservative predictions were carried out by such online software as REVEL [9], SIFT (http://sift.jcvi.org/www/SIFT_enst_submit.html), PolyPhen-2 (http://genetics.bwh.harvard.edu/pph2/), MutationTaster (http://www.mutationtaster.org/), and GERP++ (http://mendel.stanford.edu/SidowLab/downloads/gerp/index.html). The pathogenicity of each variant was calculated according to the criteria set by the American College of Medical Genetics and Genomics (ACMG) [10]. Sanger sequencing was performed on all patients and their parents in our study to confirm the presence of the identified variant loci.

### 2.3. Statistical Analysis

Continuous variables, including age at onset, age at the first visit, and age at diagnosis, were presented as median and range in box plots. Clinical features were visualized in heat map. Categorical variables were summarized by percentages and frequencies.

## 3. Results

### 3.1. Clinical Findings

**Figure 1:**
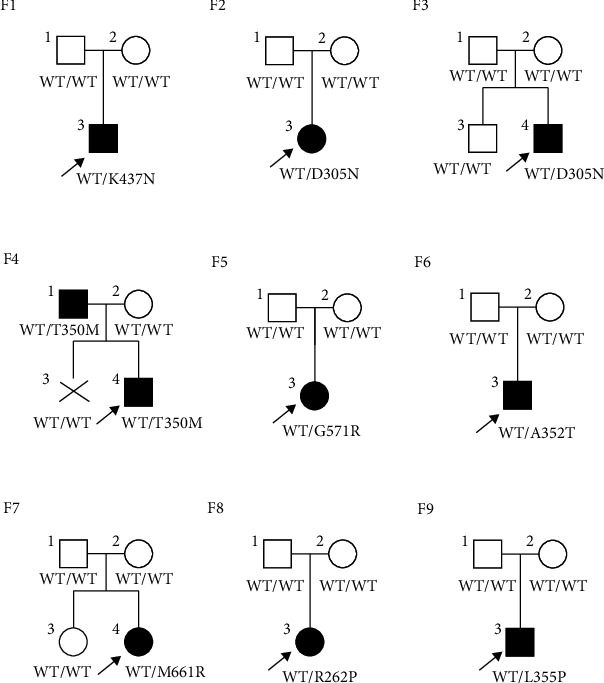
The family trees of the nine Chinese families with NLRP3-AID. Males and females were represented by squares and circles, respectively. The darkened symbol denoted that the member had been diagnosed with NLRP3-AID, and the patient above the arrow was the proband.

**Figure 2:**
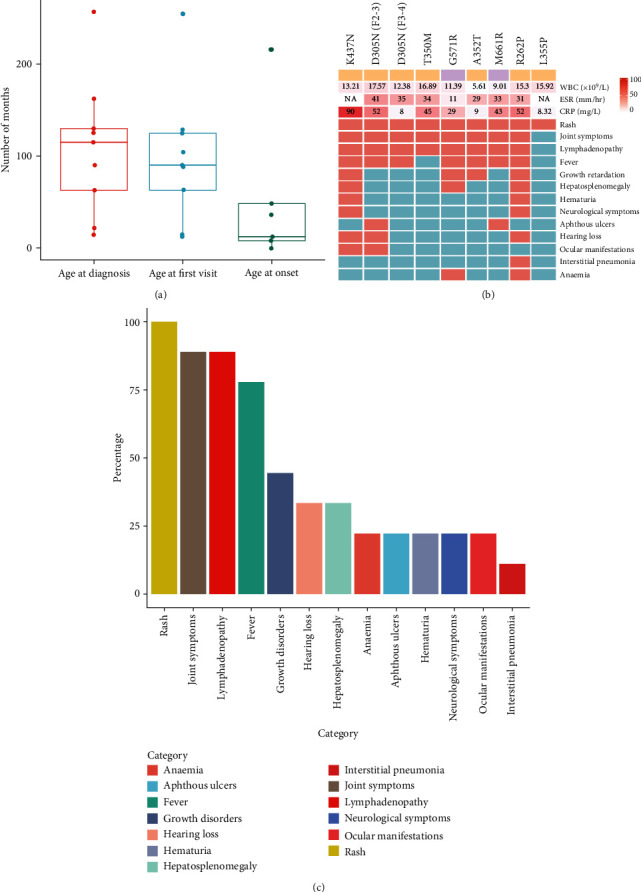
Summary data of clinical features of NLRP3-AID probands in this study. (a) Boxplot of the age distribution of the probands in this study. The findings of this study were reported using the median and interquartile range (IQR) as statistical measures. The age at the first visit was documented when the patients sought medical care at Yuying Children's Hospital of Wenzhou Medical University, Children's Hospital of Nanjing Medical University, or Children's Hospital of Soochow University. (b) Heat map of the clinical manifestations of the probands in this study. WBC, white blood cell; ESR, erythrocyte sedimentation rate; CRP, C-reaction protein; NA, not available. The horizontal axis represented amino acid changes, and amino acid changes on the same structural domain were indicated by the same color. The vertical axis represents the clinical items. The numerical type was indicated by the color depth, and the binary type was indicated by the colors red and blue, with red representing presence and blue representing absence. The increased levels of WBC, ESR, and CRP were highlighted in bold format. (c) Bar chart of the symptom incidence among the probands in this study. The height of the bar represented the occurrence rate of the symptom among the probands included in this study.

Between December 2014 and October 2022, nine patients (four females and five males) who met the inclusion criteria were recruited from Yuying Children's Hospital of Wenzhou Medical University, Children's Hospital of Nanjing Medical University or Children's Hospital of Soochow University in China, including six cases of FCAS, one case of MWS and two cases of CINCA. The average follow-up time exceeds 30 months, with five cases having follow-ups longer than 3 years. The comprehensive information about their family history provided by the participants and/or their family members is presented in Figure 1. The majority of the patients had disease onset during childhood, and six cases (66.7%) were younger than 3 years old (Table 1). The median and interquartile range (IQR) 25–75 of onset age was 8 (12, 48) months. The median and interquartile range (IQR) 25–75 of age at first visit and age at diagnosis were 90 (63, 125) months and 115 (63, 130) months, respectively (Figure 2(a)). The initial symptoms at the onset were fever (66.7%) followed by rash (66.7%) and arthralgia (44.4%). The misdiagnosis rate was as high as 44.4%, with instances involving erroneous diagnoses of septic arthritis (F1-3), adult onset still's disease (F2-3), urticaria (F9-3), and newborn sepsis and intracranial infection (F8-3). In addition, the family history was observed in one case (11.1%), as NLRP3 mutation along with rash was identified in F4-1.

**Figure 3:**
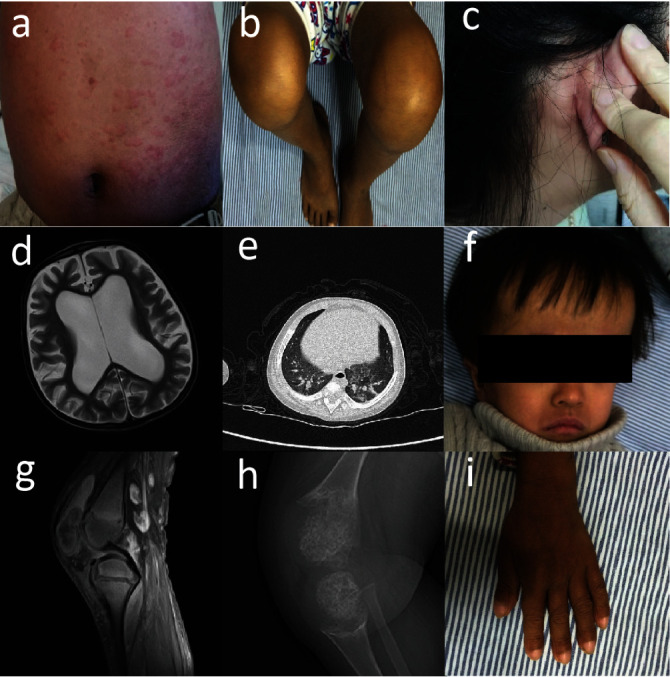
Representative photographs of the probands diagnosed as NLRP3-AID. (a) Urticarial-like rash on the abdomen of F1-3. (b) Swollen knees of F1-3. (c) Enlarged posterior auricular lymph node in F2-3. (d) Cranial magnetic resonance imaging (MRI) showed obstructive hydrocephalus and atrophy in F1-3. (e) High-resolution computed tomography (HRCT) demonstrated interstitial pneumonia in both lungs of F8-3. Scattered patchy, streaky hyperdense shadows in both lungs with blurred margins. (f) Hydrocephalus face of F1-3. (g) Knee MRI of F1-3 suggested bone destruction of the left patella, synovial hypertrophy, joint effusion, and popliteal cyst. (h) X-ray suggested right distal femur and proximal tibia stem and bone expansion in F8-3. (i) Clubbing fingers of F1-3.

As for the clinical features, increased levels of white blood cell (WBC), C-reactive protein (CRP), and/or erythrocyte sedimentation rate (ESR) had been identified in all of the patients. Notably, two or more of these elevations were observed in 88.9% of the cases. Rash, joint symptoms (including arthritis/arthralgia, swelling of the joints, and limited joint mobility), and lymphadenopathy were the most common symptoms (Figure 3(a)–3(c) and Figure 3(g)–3(h), the percentage of which were 100%, 88.9%, and 88.9%, respectively. The most frequently affected joints in this study were the knee and ankle joints, accounting for 50% and 25%, respectively. Fever occurred in 77.7% of cases (7/9), and four of them failed to obtain a normal range of inflammatory indicators 7 days or more after the resolution of the fever. Growth retardation was found in 44.4% (4/9) of the patients while anemia was only identified in two patients. Several instances of organ involvement were documented. The positive rates for hepatosplenomegaly, hematuria, aseptic meningitis, and aphthous ulcers were 33.3%, 22.2%, 22.2%, and 22.2%, respectively. The occurrence of sensorineural deafness and ocular manifestations were 33.3% and 22.2%. The eye involvement included optic papilla edema (F2-3) and suspicious retinal phlebitis (F1-3). Of noteworthy significance, atrophic hydrocephalus and clubbing fingers were both observed in two CINCA cases within this study, and the occurrence of interstitial pneumonia, a rarely reported manifestation in previous studies, was noted in one CINCA patient (F8-3) (Figures 3(d)–3(f) and 3(i). The clinical presentations are visualized in Figure 2(b) and have been summarized by the rates of positive cases in Figure 2(c).

### 3.2. Genetic Characteristics

In total, eight heterozygous missense mutations in the NLRP3 gene were screened out among those nine families, including seven known mutations and one novel mutation (Table 1). The reported mutation (c.1049C > T, p.T350M) [11], detected in patient F4-4, was inherited from the father (Figure 1). The other six known heterozygous mutations (K437N, D305N, G571R, A352T, R262P, and L355P) [11–17] were considered *de novo* mutations. The spontaneous missense mutation in NLRP3 (c.1982T > G, p.M661R of F7-4) was also identified which has never been reported in previous studies.

In order to assess the pathogenicity of these eight missense mutations, five distinct predictive software tools were employed to analyze the potential impacts of amino acid substitutions resulting from these mutations. The ultimate classification of each variant was determined following the guidelines of ACMG, resulting in categorizations of either “damaging” or “likely damaging.” Comprehensive information regarding the identified NLRP3 mutations in this study, along with corresponding pathogenic assessments, were presented in Table 1.

The genetic heterogeneity was found in this study. Three different conditions of NLRP3-AID, including FCAS, MWS, and CINCA, were all observed in variants situated on the same structural domain (K437N, D305N, T350M, A352T, R262P, and L355P). Moreover, there were variations in clinical manifestations among the six patients diagnosed with FCAS, implying different outcomes based on specific variants. As for the two cases with the identical D305N mutation, hearing loss and ocular manifestations were found exclusively in one patient (F2-3) and were not observed in the other (F3-4). As a result, the former was diagnosed with MWS, while the latter was diagnosed with FCAS. The genotype-phenotype correlations were visualized in Figure 2(b).

### 3.3. Treatment and Outcome

In this study, nonsteroidal anti-inflammatory drugs (NSAIDs), glucocorticoid (GC), thalidomide, and methotrexate were administered to 88.9% (8/9), 77.8% (7/9), 66.7% (6/9), and 55.6% (5/9) of cases, respectively. Among them, two patients used steroids in combination with thalidomide, two cases used steroids combined with methotrexate, and three used a combination of steroids, thalidomide, and methotrexate. In the use of biologics, there were two patients who received tocilizumab (22.2%, 2/9), and two patients who received canakinumab (22.2%, 2/9). Over a follow-up period ranging from 7 to 50 months for all nine cases, treatment outcomes were assessed. Notably, CR was achieved in three cases (1 FCAS and 2 MWS), PR in six cases, and no cases displayed non-remission (NR). The specific details of medication regimens were outlined in Table 1.

## 4. Discussion

This study analyzed clinical features, genotypes, and treatment responses of nine NLRP3-AID patients in China. The main clinical manifestations of NLRP3-AID in our cohort were recurrent urticaria-like rash, arthritis, and periodic fever with increased inflammatory markers. The incidence of eye and ear organ involvement was 22.2% and 33.3% separately, which was lower than the published data in other regions but consistent with reports from China. Of note, more than 60% of the patients had an onset age younger than 3 years. Inflammatory markers had not returned to normal more than 7 days after the subsiding of body temperature in more than half of the patients. Alarmingly, the misdiagnosis rate was 44.4% and the mean time of diagnosis delay was more than 5 years in our cohort, reflecting a lack of awareness regarding NLRP3-AID in China. Early diagnosis and appropriate treatment could significantly improve the quality of life, so the early identification of NLRP3-AID pediatric patients was of utmost importance.

Furthermore, some rare presentations were found in our patients. We observed interstitial lung disease (ILD) in a CINCA patient at the onset of the disease (F8-3). The patient exhibited the mutation c.785G > C, p.R262P and was diagnosed as CINCA presented with neonatal onset fever, urticaria-like rash, and hydrocephalus. Initially, the patient experienced respiratory distress after exercise, and chest high-resolution computed tomography (HRCT) revealed ILD, without clear evidence of infection. Then, he was treated with nebulized inhalation and a combination of NSAIDs and tocilizumab. Inflammatory markers partially improved, but the HRCT abnormalities persisted for several months. Therefore, steroids were added to the treatment, and tocilizumab was replaced by canakinumab, resulting in the improvement of lung imaging and respiratory symptoms. There was a study that reported a CINCA patient with the G307V mutation, who exhibited no response to anakinra and etanercept treatments. After switching to tocilizumab, the patient achieved partial clinical relief; however, the patient eventually succumbed to congestive heart failure and ILD [18]. Shu et al. [19] reported a cohort of four NLRP3-AIDs patients who presented with ILD at the onset of the disease and achieved recovery within 3–6 months of Canakinumab therapy, consistent with our report. Clubbing fingers were found in two of our CINCA patients, including the patient with ILD mentioned above, but the other patient did not exhibit any symptoms of dyspnea, and his lung CT was normal. This presentation was consistent with another clinical report in China, but it was rarely described in previous studies from other countries.

In terms of the association of phenotypes and genotypes, the patient harboring the A352T variant diagnosed as FCAS and did not have symptoms of nephrotic syndrome and hearing loss, as previously reported [14]. However, shared clinical features such as rash and fever were still present. D305N mutation was one of the most frequent NLRP3 mutations and suggested it may be associated with early disease onset, a chronic course, and hearing impairment [11, 15, 16]. It is interesting that this same variant locus resulted in two different clinical phenotypes. In our cohort, p.D305N was found in both FACS (F3-4) and MWS (F203) patients. The FCAS was definitively diagnosed at the age of 7 without hearing impairment, while the MWS patient was diagnosed at 21 with sensorineural hearing loss. Hearing loss might increase with age, indicating the need for special attention to hearing testing during follow-up in these FCAS cases. If the disease remained in continuous remission, whether hearing impairment would no longer occur in these FCAS patients still required further confirmation through long-term follow-up.

In terms of treatment, the major therapeutic approach was to block the function of IL-1 due to its central role in the pathogenic mechanism. There were three available IL-1 inhibitors as anakinra, rilonacept, and canakinumab [2], which proved to be safe and effective through numerous trials [20–25]. However, these medications were not easily accessible in mainland China. So, in our cohort, the majority of patients still received traditional disease-modifying antirheumatic drugs. However, we observed that in the biologics group, 66.7% achieved CR, without steroid treatment. Organ damage to the eyes and ears recovered to normal as disease activity was controlled, reaching a state of CR, suggesting that organ damage could partially reverse in this disease. This further emphasized the importance of early diagnosis, early treatment, and achieving standardized remission treatment strategies.

In conclusion, the most common clinical manifestations in NLRP3-AID patients were periodic fever of unknown origin, recurrent urticaria-like rash, and arthritis with increased inflammatory markers. Notably, heightened clinical vigilance was warranted when the above clinical features were present in individuals under the age of three, particularly if the inflammatory indicators failed to return to baseline levels during fever-free intervals. We also reported ILD and clubbing fingers could be found in CINCA patients, one novel pathogenic variant causing FCAS, expanding the clinical and genetic landscape of NLRP3-AID.

## Figures and Tables

**Table 1:** Clinical and genetic information of nine NLRP3-AID patients.

Patient		F1-3	F2-3	F3-4	F4-4	F5-3	F6-3	F7-4	F8-3	F9-3
Gender		M	F	M	M	F	M	F	F	M
Age (month)	Onset	0	216	48	8	48	36	12	0	12
	First visit	104	255	89	125	63	90	129	14	12
	Diagnosis	162	257	90	125	63	115	130	14	22
Exon		3	3	3	3	3	3	3	3	3
Variant	Nucleotide change	c.1311G > T	c.913G > A	c.913G > A	c.1049C > T	c.1711G > C	c.1054G > A	c.1982T > G	c.785G > C	c.1064T > C
	Amino acid	p.K437N	p.D305N	p.D305N	p.T350M	p.G571R	p.A352T	p.M661R	p.R262P	p.L355P
	Zygosity	Het	Het	Het	Het	Het	Het	Het	Het	Het
	Type	Missense	Missense	Missense	Missense	Missense	Missense	Missense	Missense	Missense
Computational prediction		DDDDDL	DDDDDD	LDDDDD	DBDDDL	.DLDBL	D.DDDD	DDBDDL	.DD..L	…..D
Novel mutation		—	—	—	—	—	—	Novel	—	—
Diagnosis		CINCA	MWS	FCAS	FCAS	FCAS	FCAS	FCAS	CINCA	FCAS
Medical regimen										
Medicine	NSAIDs	+	+	++	++	+	+	++	++	—
	Glucocorticoid	+	—	++	++	++	++	++	++	—
	Methotrexate	+	—	++	—	++	++	+	—	—
	Thalidomide	++	++	—	++	++	++	—	++	—
	Tocilizumab	—	++	—	—	—	—	—	+	—
	Colchicine	+	—	—	—	—	—	—	—	—
	Leflunomide	+	—	—	—	—	—	—	—	—
	Canakinumab	—	—	—	—	—	—	—	++	+
	Anakinra	—	—	—	—	—	—	—	—	+
The efficacy		PR	CR	PR	CR	PR	PR	PR	PR	CR

M, male; F, female; Het, heterozygous; D, damaging; B, benign; L, likely damaging; FCAS, familial cold autoinflammatory syndrome; MWS, Muckle–Wells syndrome; CINCA, chronic infantile neurologic cutaneous articular syndrome; NSAIDs, nonsteroidal anti-inflammatory drug; PR, partial remission; and CR, complete remission. Computational prediction: Letters represent the prediction of REVEL, SIFT, Polyphen-2, MutationTaster, GERP+, and final classification (American College of Medical Genetics and Genomics) in turn. The dot represented the lack of prediction results in this way. The medication regimen after the detection of the gene mutation was marked once more on the basis of the medication applied before the gene mutation seizure, and there were no new drugs after the seizure. + represents past treatment medication, and ++ represents current treatment medication.

## Data Availability

The original data presented in the study were included in this article. Further inquiries could be directed to the corresponding author.

## References

[1] Booshehri L. M., Hoffman H. M. (2019). CAPS and NLRP3. *Journal of Clinical Immunology*.

[2] Kuemmerle-Deschner J. B. (2015). CAPS—pathogenesis, presentation and treatment of an autoinflammatory disease. *Seminars in Immunopathology*.

[3] Hoffman H. M., Wanderer A. A., Broide D. H. (2001). Familial cold autoinflammatory syndrome: phenotype and genotype of an autosomal dominant periodic fever. *The Journal of Allergy and Clinical Immunology*.

[4] Muckle T. J., Wells M. (1962). Urticaria, deafness, and amyloidosis: a new heredo-familial syndrome. *The Quarterly Journal of Medicine*.

[5] Prieur A. M., Griscelli C. (1981). Arthropathy with rash, chronic meningitis, eye lesions, and mental retardation. *The Journal of Pediatrics*.

[6] Wu Z., Wu S., Liang T. (2021). Association of NLRP3 rs35829419 and rs10754558 polymorphisms with risks of autoimmune diseases: a systematic review and meta-analysis. *Frontiers in Genetics*.

[7] Hoffman H. M., Mueller J. L., Broide D. H., Wanderer A. A., Kolodner R. D. (2001). Mutation of a new gene encoding a putative pyrin-like protein causes familial cold autoinflammatory syndrome and Muckle–Wells syndrome. *Nature Genetics*.

[8] Gattorno M., Hofer M., Federici S. (2019). Eurofever R, the paediatric rheumatology international trials O: classification criteria for autoinflammatory recurrent fevers. *Annals of the Rheumatic Diseases*.

[9] Ioannidis N. M., Rothstein J. H., Pejaver V. (2016). REVEL: an ensemble method for predicting the pathogenicity of rare missense variants. *American Journal of Human Genetics*.

[10] Richards S., Aziz N., Bale S. (2015). Committee ALQA: standards and guidelines for the interpretation of sequence variants: a joint consensus recommendation of the American College of Medical Genetics and Genomics and the association for molecular pathology. *Genetics in Medicine*.

[11] Dode C., Le Du N., Cuisset L. (2002). New mutations of CIAS1 that are responsible for Muckle–Wells syndrome and familial cold urticaria: a novel mutation underlies both syndromes. *American Journal of Human Genetics*.

[12] Wang W., Yu Z., Gou L. (2020). Single-center overview of pediatric monogenic autoinflammatory diseases in the past decade: a summary and beyond. *Frontiers in Immunology*.

[13] Zhou Y., Wang W., Zhong L. (2022). Clinical and genetic spectrum of 14 cases of NLRP3-associated autoinflammatory disease (NLRP3-AID) in China and a review of the literature. *Orphanet Journal of Rare Diseases*.

[14] Rowczenio D. M., Gomes S. M., Arostegui J. I. (2017). Late-onset cryopyrin-associated periodic syndromes caused by somatic NLRP3 Mosaicism-UK single center experience. *Frontiers in Immunology*.

[15] Li C., Tan X., Zhang J. (2017). Gene mutations and clinical phenotypes in 15 Chinese children with cryopyrin-associated periodic syndrome (CAPS). *Science China Life Sciences*.

[16] Louvrier C., Assrawi E., El Khouri E. (2020). NLRP3-associated autoinflammatory diseases: phenotypic and molecular characteristics of germline versus somatic mutations. *The Journal of Allergy and Clinical Immunology*.

[17] Hoffman H. M., Gregory S. G., Mueller J. L. (2003). Fine structure mapping of CIAS1: identification of an ancestral haplotype and a common FCAS mutation, L353P. *Human Genetics*.

[18] Matsubara T., Hasegawa M., Shiraishi M. (2006). A severe case of chronic infantile neurologic, cutaneous, articular syndrome treated with biologic agents. *Arthritis and Rheumatism*.

[19] Shu Z., Zhang Y., Han T. (2023). The genetic and clinical characteristics and effects of Canakinumab on cryopyrin-associated periodic syndrome: a large pediatric cohort study from China. *Frontiers in Immunology*.

[20] Hawkins P. N., Lachmann H. J., McDermott M. F. (2003). Interleukin-1-receptor antagonist in the Muckle–Wells syndrome. *The New England Journal of Medicine*.

[21] Alexander T., Klotz O., Feist E., Ruther K., Burmester G. R., Pleyer U. (2005). Successful treatment of acute visual loss in Muckle–Wells syndrome with interleukin 1 receptor antagonist. *Annals of the Rheumatic Diseases*.

[22] Sibley C. H., Plass N., Snow J. (2012). Sustained response and prevention of damage progression in patients with neonatal-onset multisystem inflammatory disease treated with anakinra: a cohort study to determine three- and five-year outcomes. *Arthritis and Rheumatism*.

[23] Hoffman H. M. (2009). Rilonacept for the treatment of cryopyrin-associated periodic syndromes (CAPS). *Expert Opinion on Biological Therapy*.

[24] Stahl N., Radin A., Mellis S. (2009). Rilonacept—CAPS and beyond. *Annals of the New York Academy of Sciences*.

[25] Lachmann H. J., Kone-Paut I., Kuemmerle-Deschner J. B. (2009). Canakinumab in CSG: use of canakinumab in the cryopyrin-associated periodic syndrome. *The New England Journal of Medicine*.

